# MRI and CT Appearances in Various Cardiac Tumours

**DOI:** 10.7759/cureus.51488

**Published:** 2024-01-01

**Authors:** Aayush Chauhan, Abhay Mudey, Harshit Singh

**Affiliations:** 1 Radiology, Jawaharlal Nehru Medical College, Datta Meghe Institute of Higher Education and Research, Wardha, IND; 2 Community Medicine, Jawaharlal Nehru Medical College, Datta Meghe Institute of Higher Education and Research, Wardha, IND; 3 Pathology, Jawaharlal Nehru Medical College, Datta Meghe Institute of Higher Education and Research, Wardha, IND

**Keywords:** tumour, computed tomography, electrocardiography, magnetic resonance, malignancy

## Abstract

While primary cardiac malignancies are infrequent, the heart often serves as a site for metastases. Myxomas are recognized as among the most prevalent primary benign tumours globally, while sarcomas represent the most common malignant primary tumours. The diverse range of potential clinical presentations depends on factors such as location, size, and the aggressiveness of the disease. The majority of diagnoses rely on medical imaging, making it crucial to familiarize oneself with their distinctive characteristics. When a cardiac mass is suspected, MRI of the heart has emerged as the preferred diagnostic method, surpassing previous techniques.

CT is a valuable tool for assessing cardiac morphology and improving electrocardiography gating by providing enhanced details. This article conducts a comprehensive review of the MRI and CT characteristics of both primary and secondary cardiac malignancies, emphasizing crucial distinctions and common diagnostic pitfalls. Despite their rarity, cardiac masses continue to hold significance in the realm of cardio-oncology. Furthermore, this article explores conditions such as thrombus, Lambl's excrescences, and pericardial cysts, which can mimic tumours. Multimodal imaging has played a pivotal role in identifying the origin of cardiac masses in numerous cases, particularly when combined with the clinical context. This article offers an in-depth examination of the frequency, clinical indicators, imaging, diagnostic procedures, available treatments, and prognoses related to cardiac masses.

## Introduction and background

According to autopsy estimates, benign and malignant primary cardiac tumours occur in approximately 0.002% to 0.3% of cases [1]. Primary benign cardiac tumours encompass myxomas, fibromas, rhabdomyomas, lipomas, fibroelastomas, hemangiomas, and paragangliomas. The three most frequently occurring primary malignant cardiac neoplasms are lymphomas, mesotheliomas, and sarcomas. Secondary cardiac tumours, also known as cardiac metastases, are more common (20%-40%) and generally have a poor prognosis due to the advanced stage of the underlying malignancy from which they originate [2]. Pseudotumors, or tumour-like structures, are much more common than actual tumours. Examples include intracardiac thrombus, pericardial cysts, valve vegetations, perivalvular abscesses, and normal cardiac variations like the crista terminalis.

Patients who are suffering from primary cardiac tumours may experience symptoms often misinterpreted as signs of heart disease. The critical factors in determining a tumour's clinical presentation are its location, size, texture, growth rate, and invasiveness [3]. Mechanical obstruction resulting from a cardiac mass in the outflow tract can lead to symptoms such as heart failure, valve disease, and systolic and diastolic dysfunctions due to reduced contractility.

Various imaging techniques are employed to evaluate cardiac and pericardial tumours, each with its advantages and drawbacks. Transthoracic echocardiography has become the standard method for detecting intracardiac tumours due to its widespread availability. However, it provides limited information about the tissue characteristics of the mass, beyond its size, motion, shape, and location in the heart. Transesophageal echocardiography may be used in diagnosing certain individuals, such as those who are overweight or have chronic obstructive pulmonary disease [4].

Cardiac CT scans are non-invasive procedures that provide precise images in both spatial and temporal dimensions. Although CT imaging can clearly depict cardiac and intracardiac masses, as well as assess the extent of myocardial and pericardial involvement, it has limitations, including radiation exposure and the requirement for iodinated contrast injection [5].

Cardiac magnetic resonance (CMR) offers several advantages for diagnosing individuals with heart tumours. Additionally, 18-fluoro-deoxyglucose positron emission tomography (FDG PET) is an effective technique for determining tumour recurrence, treatment response, biopsy site selection, radiation therapy planning, and disease staging [6]. The tumours can be categorized as benign or malignant based on FDG uptake [7].

Utilizing CMR image-based motion correction improves spatial and temporal alignment in combined positron emission tomography (PET)-MRI systems, reducing motion artefacts such as blurring and partial volume effects in PET scans [8]. A combined PET-MRI system can significantly reduce data acquisition time, enhance throughput, and minimize patient discomfort compared to conducting two separate tests [9].

## Review

Methodology

**Figure 1:**
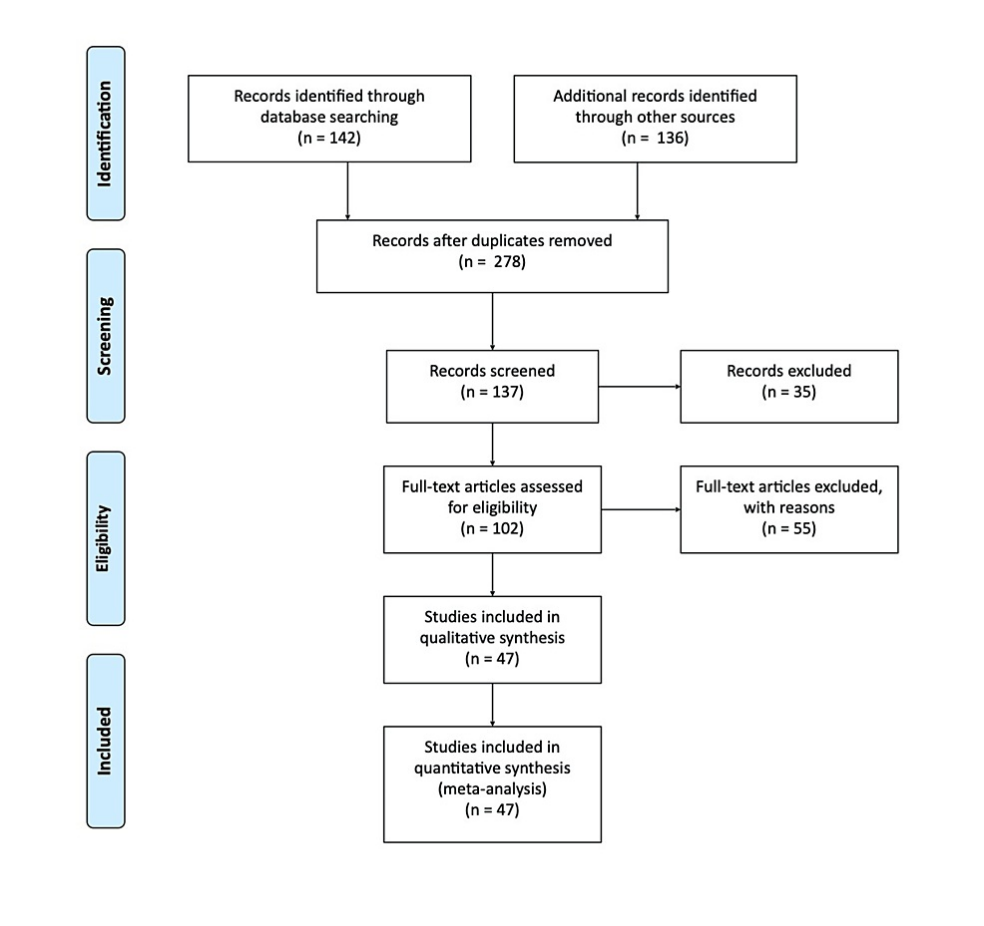
Preferred Reporting Items for Systematic Reviews and Meta-Analyses (PRISMA) methodology

This extensive study used a methodical way to compile and examine pertinent research on MRI and CT appearances in different cardiac tumours. To achieve a thorough grasp of the issue, the study included both qualitative and quantitative research studies and academic articles. To find pertinent materials, a thorough search was carried out across many academic databases, including PubMed, Scopus, and Google Scholar. In a database like PubMed, relevant terms and phrases like "echocardiography", "magnetic resonance", "radiation", "malignancy", and "tumour" were searched for. There were only English-related results shown. The most recent report from a similar study was utilized if there were multiple published reports. We only considered review publications that also included original data. Figure 1 shows the search strategy utilized for the review. The articles which were in English language and published before June 2023 were only included in the review. The articles in other languages were excluded. 

Benign cardiac tumours versus malignant cardiac tumours: differentiating features

Certain cardiac MRI findings can aid in distinguishing between benign and malignant heart tumours. Factors such as size, location, and the presence of calcification in a pericardial effusion are all important considerations. Typically, most cardiac tumours are benign, characterized by a well-defined border [10]. In contrast, benign cardiovascular tumours often have much bigger bases. Benign tumours are usually considerably smaller in size than malignant ones, with some malignant tumours being so large that they nearly occupy an entire chamber [11]. Given the rarity of benign tumours on the right atrial wall, the presence of a cardiac tumour in this location should raise suspicion of malignancy [12]. Cardiac osteosarcoma often exhibits extensive calcification, while benign tumours like myxomas and fibromas may show smaller areas of calcification. When both a heart tumour and pericardial effusion are present, the possibility of malignancy should be considered [13].

Differentiating Primary and Malignant Cardiac Tumors From Thrombus

Detection of thrombus is a significant contributing factor for primary and malignant cardiac tumours. Finding a heart thrombus is frequently considered essential for stopping emboli and providing a solid foundation for a medication called an anticoagulant. Cardiac MRI has emerged as a superior imaging modality compared to traditional echocardiography, which has long been the gold standard for identifying thrombi [14]. The incidence of thrombi can vary significantly between examinations. Contrast-enhanced cardiac MRI is particularly effective in distinguishing thrombi from the surrounding myocardium since thrombi, being avascular entities, do not absorb contrast agents [15]. However, it's worth noting that massive chronic thrombi can occasionally extend outward and become challenging to visualize [16].

In a study focused on cardiac thrombus detection using MRI, 15 thrombi were identified compared to only 12 thrombi detected by echocardiography. Areas of low signal intensity were indicative of the presence of thrombi, representing filling defects within the cardiac cavities. Another advantage of delayed-enhanced cardiac MRI sequences is their ability to differentiate between tumours and thrombi [17]. Delayed-enhanced cardiac MRI sequences were employed to identify myocardial thrombi in 779 consecutive high-risk patients with systolic dysfunction [18]. Thrombi were detected in 7% of patients (n = 55) using delayed-enhanced cardiac MRI, whereas only 4.7% of patients (n = 37) were detected using cine cardiac MRI [19].

Fundamental Cardiac MRI Procedures for Evaluating Heart Tumors

**Figure 2:**
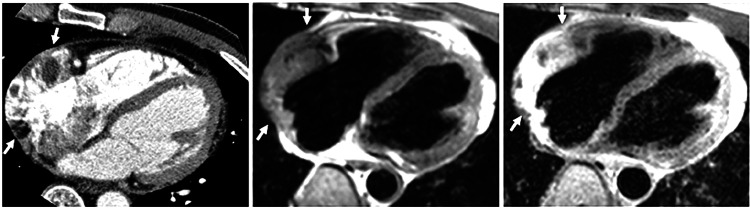
Typical cardiac MRI sequences that are used to assess heart tumours. A 70-year-old lady who is developing dyspnea. The axial T1-weighted pictures show uneven enlargement of the right atrial mass before and after gadolinium administration. Right atrial myxoma was discovered during surgery. (A) CT scan with an ECG gated multidetector reveals a big mass (arrows) in the right atrium's free wall. The mass has a nodular, erratic shape with significant contrast enhancement. (B) Recovery from transverse double inversion. The right atrium's isointense mass (arrows) is largely visible in the MR picture. (C) Recovery after transverse triple inversion. A heterogeneously hyperintense mass is shown in the MR picture (arrows). Image Credit: Kim et al., 2009 [21]; Licensed under CC-BY

It is imperative to tailor the cardiac MRI technique to the specific characteristics of the tumour being imaged. We have found that having a radiologist present for the initial image review is crucial to avoid using standard imaging procedures and image planes that may not accurately depict the mass. Although there are several available cardiac MRI sequences, they all follow the same fundamental structure [20]. The primary objective when imaging cardiac tumours is to define the mass's location, size, and extent. In the following sections, we will delve into the fundamental MRI sequences used for the assessment of cardiac tumours. Technical specifications for our imaging were based on Magnetom Avanto 1.5T scanner (Siemens Healthineers, Erlangen, Germany) (Figure 2).

Benign cardiac tumors

Cardiac Myxoma

**Figure 3:**
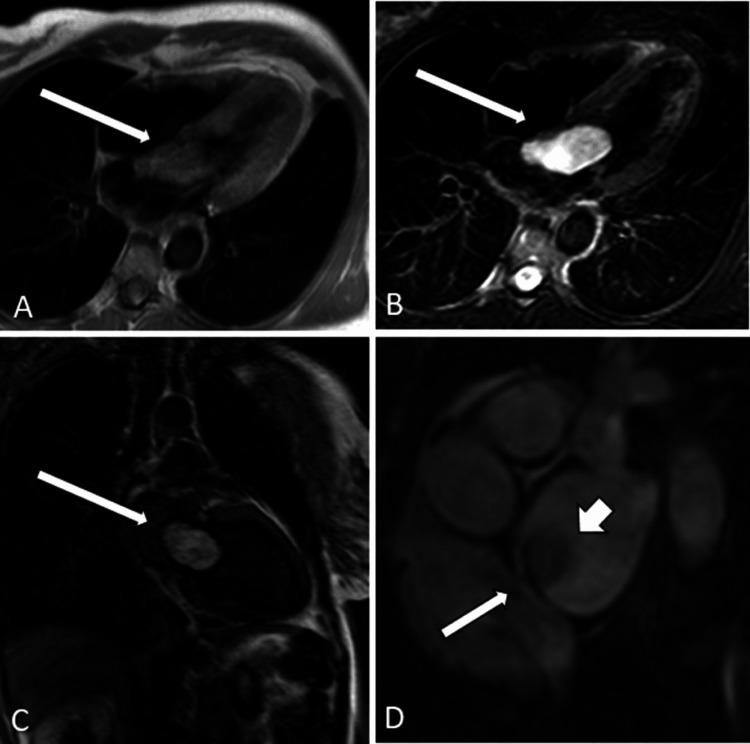
(A) A T1-weighted horizontal four-chamber image revealing a mass with mostly isointense signal (arrow). (B) The mass is properly distributed, and the strength of the signal is very strong along (arrow) in this T2-weighted horizontal four-chamber image. (C) A significant amount of heterogeneous enhancement can be seen throughout the mass (arrow). (D) An oblique gradient echo (GRE) image illustrating the relationship between the mass and the patent foramen ovale (arrow). Image Credit: Grant et al., 2017 [24]; Licensed under CC-BY

A rare benign tumour that can develop in the heart is known as cardiac myxoma. These tumours are most commonly found in one of the heart's chambers, particularly the left atrium. Myxomas often consist of stellate cells and connective tissue [22-24]. Cardiac myxomas often develop slowly, and in some cases, they can impede blood flow through the heart chambers or cause problems with the heart valves (Figure 3).

Symptoms of a cardiac myxoma may include shortness of breath, fatigue, dizziness, irregular heartbeats (arrhythmias), and stroke-like symptoms if a fragment of the tumour breaks off and travels to the brain (embolism) [23].

The exact cause of cardiac myxomas is not fully understood. Typically, the best treatment option is by performing surgery. Thanks to advancements in medical imaging and surgical techniques, the prognosis for individuals with cardiac myxomas has improved. If you suspect that you or someone you know is experiencing symptoms related to the heart, it is crucial to seek medical attention for a comprehensive evaluation and appropriate recommendations [25].

Lipoma and Lipomatous Hypertrophy of the Interatrial Septum (LASH)

LASH is a benign condition characterized by a significant accumulation of fat, typically exceeding 2 cm in thickness, within the interatrial septum [26]. This condition is commonly observed in older individuals and those who are overweight. Unlike true lipomas, LASH lacks a capsule and comprises a combination of lipoblasts and mature fat cells [27]. In some cases, increased radiotracer uptake on PET/CT scans can be attributed to brown fat in the affected area [28]. To confirm the diagnosis of LASH, the fat pad should be thicker than 20 mm and exhibit a bilobed dumbbell shape, sparing the fossa ovalis [29]. Symptoms associated with this condition are rare, although atrial arrhythmias have been reported in some cases [30].

Papillary fibroelastomas

**Figure 4:**
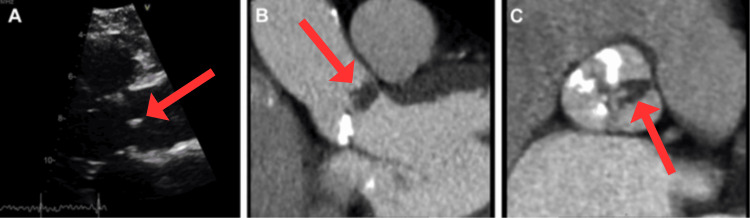
Papillary fibroelastoma Image Credit: Tyabelly et al., 2020 [33]; Licensed under CC-BY

Papillary fibroelastomas (Figure 4) are among the most common types of primary cardiac tumours. With the advancement of spatial resolution in imaging modalities, they are increasingly recognized. Additionally, they can be a contributing factor to conditions such as strokes, myocardial infarction, transient ischemic attacks, and sudden death [31]. While papillary fibroelastomas and cardiac myxomas are typically benign, other primary cardiac tumours can have varying degrees of malignancy and clinical significance. Appropriate diagnosis and treatment planning are crucial for managing these cardiac tumours. Haematological features, location within the heart, size, and aggressiveness are the factors that differentiate papillary fibroelastomas, cardiac myxomas, and other primary cardiac tumours [32].

Fibroma of the Heart

**Figure 5:**
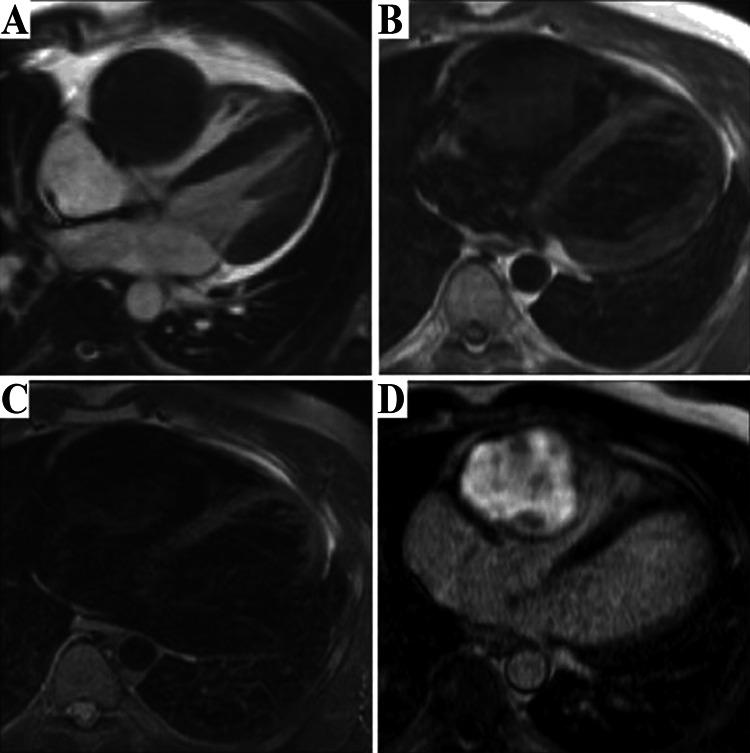
Cardiac Fibromas (A), with little signal on spin-echo images (B) and T two-weighted (C). The late phase following gadolinium injection shows both diffuse and strong amplification, with some core regions showing comparatively low signal (D). Image Credit: Teis et al., 2011 [36]; Licensed under CC-BY

Cardiac fibroma is the second most common type of congenital tumour, and it can affect individuals ranging from infants to adolescents and even young adults (Figure 5). In rare cases, cardiac fibromas may be associated with polyposis syndromes such as Gardner's syndrome and familial adenomatous polyposis [34]. These tumours typically originate in the interventricular septum and tend to have a greater impact on the left ventricle compared to the right [35].

Rhabdomyoma

**Figure 6:**
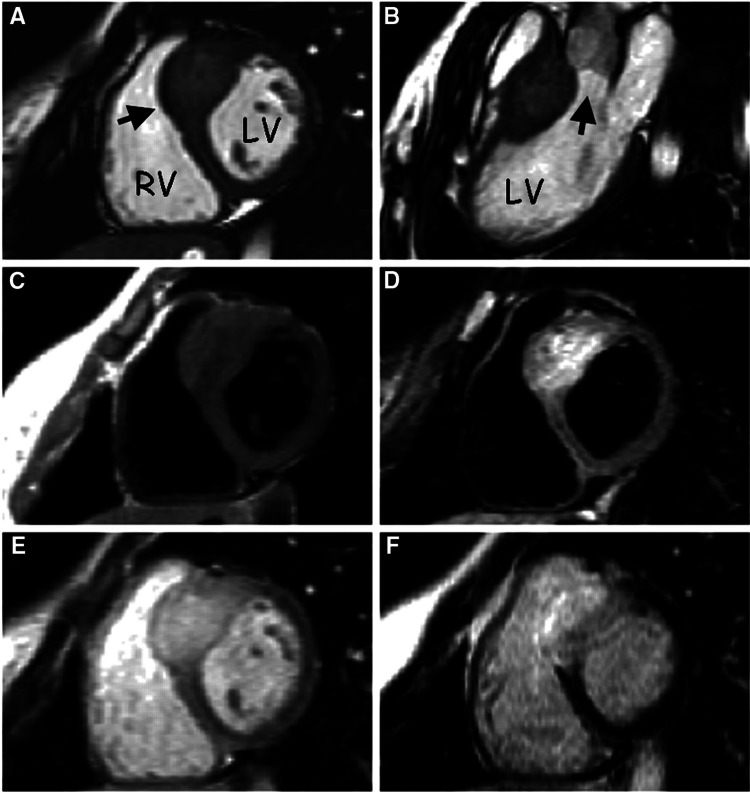
(A) A constant state, short-axis spontaneous precession movie captured through the distal septum. With the exception of the pronouncedly enlarged membrane (arrow), the left ventricle (LV) and the right ventricle (RV) appear to have the same heart muscle. A steady-state free precession video's still image (B) displays the LV exit channel (arrow). (C) The septum's signal is isointense compared to the rest of the ventricular myocardium. (D) A hyperintense signal is seen around the enlarged septum, demonstrating that it is an independent structure from the neighbouring myocardial region. (E) Early gadolinium enhancement of the myocardium, including the thicker septum, and the LV and RV cavities in a short-axis picture. (F) A short-axis picture taken following the injection of gadolinium. Normally, the myocardium is dark. The section of the enlarged septum has a clear anomalous late enhancement throughout. Image Credit: Wage et al., 2008 [38]; Licensed under CC-BY

Cardiac rhabdomyoma is a rare tumour composed of striated muscles. It is a unique type of hamartoma that primarily affects children (Figure 6). While it is typically asymptomatic, some children may exhibit symptoms of congestive heart failure. Arrhythmias can also occur if the tumour affects the conduction pathways. In cases of hemodynamic compromise and disease progression, medical intervention and surgical resection may be necessary [37].

Hemangioma of the Heart

Tumours arising from the left atrial (LA) wall and being initially mistaken for myxomas are exceptionally rare, as is the case with cardiac hemangiomas. In one instance, we removed a mass that was initially thought to be a myxoma, but subsequent pathology revealed that it was, in fact, a cardiac capillary hemangioma. Such occurrences are indeed quite uncommon.

Primary malignant tumours of the heart

Angiosarcoma

**Figure 7:**
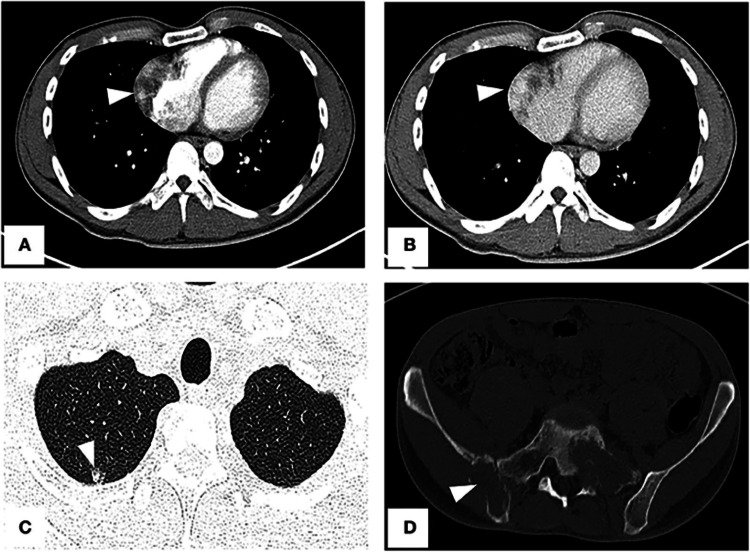
The right atrial tumour, a little ground-glass pulmonary nodule, and bone destructions are seen on an enhanced CT scan of the chest and pelvis. (A) Right atrium filling deficiency is shown on arterial phase imaging (arrowhead). Venous phase imaging (B) reveals the tumour's uneven enhancement (arrowhead). (C) A metastatic nodule can be seen in the upper right lung on a lung window image. (D) Destroyed bones in the sacrum and right ilium. Image Credit: Li et al., 2022 [39]; Licensed under CC-BY

Angiosarcoma (Figure 7) is an aggressive and malignant tumour of endothelial cell origin, often originating from lymphatic or vascular tissues. This type of cancer frequently exhibits both local recurrence and metastasis. Angiosarcoma represents less than 1% of all soft-tissue sarcomas.

There are two primary morphological classes of angiosarcomas. The occurrence of conditions like tamponade and right-sided heart failure in individuals with angiosarcomas is directly linked to the location of the tumours. Unfortunately, the prognosis is bleak because of the high prevalence (66-89%) of preexisting metastases at the time of diagnosis. T1-weighted fast spin echo (FSE) sequences often reveal low signal intensity in these tumours [40]. The administration of IV gadolinium leads to a significant signal enhancement due to the tumours' high vascularity.

Undifferentiated Sarcoma

The majority of undifferentiated sarcomas are typically located in the left atrium of the heart. They can present as either a discrete mass or something more infiltrative and irregular, often accompanied by areas of necrosis and haemorrhage. FSE images of the heart frequently reveal isointense contrast between the tumour and the myocardium. Recent research indicates that approximately 24% of primary cardiac malignancies are attributed to undifferentiated sarcomas [41]. Unfortunately, the prognosis for both adult angiosarcomas and undifferentiated sarcomas is generally unfavourable.

Fibrosarcoma

Fibrosarcoma is a rare, malignant (cancerous) type of soft tissue sarcoma. It tends to affect patients between the ages of 10 to 15 and, in some cases, infants and small children under the age of five. Fibrosarcoma typically develops in areas of the body with fibrous tissues, much like other sarcomas. It often involves the deeper tissues and extremities of the body.

Liposarcoma

Liposarcomas represent the most common subtype of soft tissue sarcomas, accounting for at least 20% of all adult sarcomas. Soft tissue sarcomas are a group of rare neoplasms characterized by over 150 distinct histological subtypes. Liposarcomas originate from the progenitor lipoblasts, which are the precursor cells of adipocytes or fat cells in adipose (fat) tissues. Adipose tissues are distributed throughout the body, including in areas like the deeper and more superficial layers of subcutaneous tissues, as well as less accessible locations such as the retroperitoneum (the area behind the abdominal cavity) and visceral fat within the abdominal cavity [42].

Extraskeletal Cardiac Osteosarcoma

Cardiac osteosarcomas represent approximately 4-10% of primary malignant heart tumours [43]. They tend to be localized in the left atrium and are often associated with symptoms of pulmonary congestion. One of the most prominent features when noninvasive imaging is performed on these lesions is the presence of substantial calcification. Cardiac MRI can sometimes make it challenging to detect the presence of intratumoral calcification, whereas CT scans can offer valuable contextual information.

These tumours have the ability to firmly attach to the host tissue and aggressively invade the regional circulatory systems. Histologically speaking, they may contain chondroblast elements and in some cases fibrous elements in addition to their mostly osteoblastic composition. Unfortunately, the prognosis for individuals with cardiac osteosarcomas is generally quite bleak across all categories.

Primary Cardiac Lymphoma

Primary cardiac lymphoma is a relatively rare occurrence compared to diffuse non-Hodgkin's lymphoma, which affects the heart less frequently. Patients with compromised immune systems are typically at a higher risk of developing aggressive B-cell lymphomas that originate as primary cardiac lymphomas. A more favourable prognosis can often be achieved with early diagnosis and prompt initiation of chemotherapeutic treatment.

It's common to observe significant pericardial effusion alongside these tumours, which typically form in the right atrium of the heart. In some cases, an MRI scan may only show the presence of a pericardial effusion. When these tumours first manifest, they often present with multiple nodules or extensive infiltration of the right ventricle [44].

Cardiac metastases

Cardiac metastasis occurs when cancerous cells from another organ, such as the liver, lungs, or breasts, travel to the heart through the bloodstream or lymphatic system. Cardiac metastases are more common compared to primary heart tumours.

**Figure 8:**
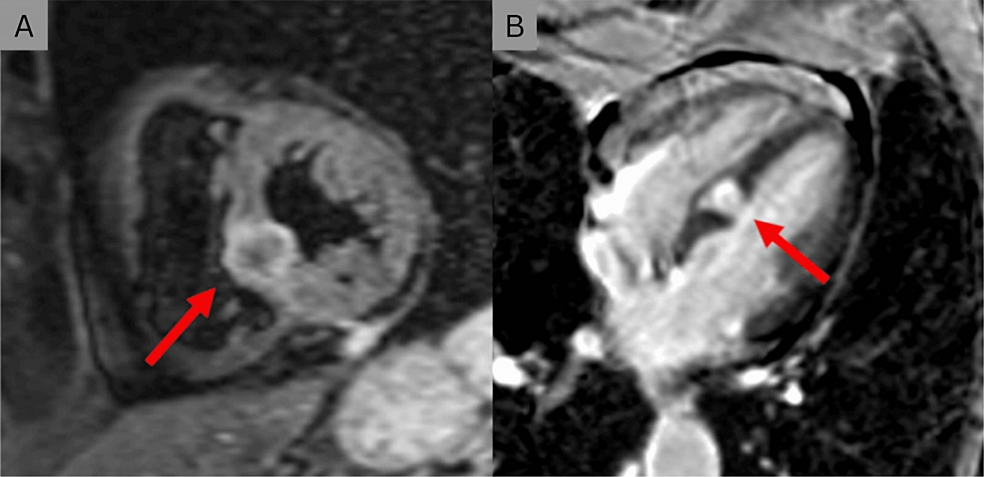
(A) Cardiac MRI scan in sagittal view may reveal a highly intense tumour within the septum between the ventricular chambers (red arrow). (B) After gadolinium injection, an axial cardiac MRI scan shows a granular improvement inside the interventricular septum (red arrow). Image Credit: Jumeau et al., 2020 [45]; Licensed under CC-BY

Symptoms of cardiac metastases can include shortness of breath, chest discomfort, irregular heartbeats, fluid retention, and fatigue. Diagnosis can be challenging because these symptoms can resemble those of other cardiac conditions (Figure 8).

The treatment approach for cardiac metastasis depends on several factors, including the underlying cancer type, the extent of metastasis, and the overall condition of the patient [46]. Treatment strategies may include a combination of surgery, radiation therapy, chemotherapy, targeted therapies, and palliative care, with the goal of relieving symptoms and improving the patient's quality of life [47].

Cardiac MRI has rapidly become a standard practice in cardiac imaging due to recent technological advancements. It has emerged as a powerful tool for diagnosing heart cancer, which is particularly crucial given the potential impact of these tumours on cardiac hemodynamics and their role as substrates for arrhythmias. Despite their rarity, some cardiac tumours can be malignant, making their early and accurate diagnosis crucial.

Cardiac MRI offers several advantages, including an expanded field of view, enhanced soft-tissue depiction without the use of ionizing radiation, and the ability to provide detailed tumour characterization. When performed correctly, it allows for the most precise description of cardiac tumour architecture and tissue characteristics, as well as the most accurate assessment of their functional impact.

## Conclusions

Life-threatening complications, such as intracardiac obstruction and fatal arrhythmias, can arise even from benign cardiac tumours. Despite their rarity, cardiac tumours carry significant clinical implications. The increased availability of multimodality imaging allows us to more accurately distinguish between different types of cardiac masses and provide the most effective medical therapy to our patients. The findings from imaging studies not only help identify the likely cause but also guide further diagnostic investigations and the development of treatment plans, which may involve medical, surgical, or combined approaches.
